# The phenomenology of auditory verbal hallucinations in schizophrenia and the challenge from pseudohallucinations

**DOI:** 10.3389/fpsyt.2022.826654

**Published:** 2022-08-16

**Authors:** Pablo López-Silva, Álvaro Cavieres, Clara Humpston

**Affiliations:** ^1^School of Psychology, Faculty of Social Sciences, Universidad de Valparaíso, Valparaíso, Chile; ^2^Millennium Institute for Research in Depression and Personality (MIDAP), Santiago, Chile; ^3^Department of Psychiatry, School of Medicine, Faculty of Medicine, Universidad de Valparaíso, Valparaíso, Chile; ^4^School of Psychology, University of York, York, United Kingdom; ^5^School of Psychology, Institute for Mental Health, University of Birmingham, Birmingham, United Kingdom

**Keywords:** pseudohallucinations, phenomenology, schizophrenia, auditory verbal hallucinations, neurobiology

## Abstract

In trying to make sense of the extensive phenomenological variation of first-personal reports on auditory verbal hallucinations, the concept of pseudohallucination is originally introduced to designate any hallucinatory-like phenomena not exhibiting some of the paradigmatic features of “genuine” hallucinations. After its introduction, Karl Jaspers locates the notion of pseudohallucinations into the auditory domain, appealing to a distinction between hallucinatory voices heard within the subjective *inner* space (pseudohallucination) and voices heard in the *outer* external space (real hallucinations) with differences in their sensory richness. Jaspers' characterization of the term has been the target of a number of phenomenological, conceptual and empirically-based criticisms. From this latter point of view, it has been claimed that the concept cannot capture *distinct* phenomena at the neurobiological level. Over the last years, the notion of pseudohallucination seems to be falling into disuse as no major diagnostic system seems to refer to it. In this paper, we propose that even if the concept of pseudohallucination is not helpful to differentiate *distinct* phenomena at the neurobiological level, the inner/outer distinction highlighted by Jaspers' characterization of the term still remains an open explanatory challenge for dominant theories about the neurocognitive origin of auditory verbal hallucinations. We call this, “*the challenge from pseudohallucinations*”. After exploring this issue in detail, we propose some phenomenological, conceptual, and empirical paths for future research that might help to build up a more contextualized and dynamic view of auditory verbal hallucinatory phenomena.

## Pseudohallucinations and phenomenological diversity of auditory verbal hallucinations

Hallucinatory phenomena are common symptoms of a number of psychiatric conditions [see (1–3)]. Although hallucinatory phenomena vary in form and might involve different sensory modalities, the presence of *hallucinations* reported as *voices*—namely, Auditory Verbal Hallucinations (AVHs henceforth)—has been historically regarded as particularly relevant to establishing schizophrenia's specific diagnosis [see (4–8)]. In order to establish a good quality differential diagnosis (e.g., AVHs in grief, trauma, borderline personality disorders, psychosis, among many others), AVHs are usually assessed in terms of reality testing (preserved/altered), context of emergence (understanding or explanation), and temporality (acute or transient vs. chronic or recurrent). From a descriptive point of view, people diagnosed with schizophrenia report hearing voices with multiple characteristics [see (9–15)]. They report being addressed by the hallucinatory voices from different perspectives (2nd or 3rd person), or by a number of different entities over time (13, 16–18). For example, an individual with schizophrenia diagnosis reports: “I hear distinct voices. Each voice has their own personality […] Many of them have identified themselves and given themselves names” [(13), p. 325]; another person reports: “I hear a mixture of men and women, but no children” (p. 325). The content and quality of voices also vary. In terms of content, while some voices are described as helpful (*ego-syntonic nature*) or non-problematic, others are reported as derogatory and menacing (*ego-dystonic nature*) (1, 10, 19). While a patient reports “I heard the voices of demons screaming at me, telling me I was damned, that God hated me” [(13), p. 326], another patient reports “They usually tell me to do things, but not dangerous things. Like they'll tell me to take out the garbage or check the lock on the window” [(13), p. 325]. The majority of voices have an identifiable gender, with male voices being more common than female ones (20, 21). Addressing the predominantly ego-dystonic nature of voices, Kraepelin (19) also observes that “many of the voices make remarks about thoughts and the doing of the patients” (p. 10), thus stressing the way in which the phenomenon is involved in the patients' ongoing everyday life's activities. In terms of quality, some voices consist of loose words, while others are identified as complete sentences. While some voices might have a distinguishable content, most of them are also heard against a background of different noise-like sounds (10, 15, 19). Sometimes voices might be heard as soft whispers or loudly like screams, while other voices are reported as devoid of sensorial qualities (soundless voices) or with a quality close to normal hearing [cf. (12, 13, 22)].

As clearly noted, there are extensive phenomenological variations in the first-person reports of AVHs, making them highly heterogenous phenomena (23, 24). Within the history of psychiatry, the term *pseudohallucination* (in a strict sense, “false hallucination”) is originally introduced in the context of European Psychiatry (especially *French* and *German*) to designate any hallucinatory-like phenomena not exhibiting the paradigmatic features of *genuine* hallucinations (7, 25–28). After its introduction, Karl Jaspers locates the notion of pseudohallucination into the auditory domain, conferring it semiological specificity. Over the years, a number of criteria have been proposed to distinguish between genuine AVHs and auditory pseudohallucination [for a review of this issue, see (2)]. However, most of them have been systematically criticized due to their insufficient diagnostic value, conceptual problems, and lack of strong empirical support [see (26, 28, 29)]. The main criticism in this context is that the term is not able to discriminate between different phenomena at the neurobiological level so, arguably, hallucinations and what it is usually called pseudohallucination might belong in a continuum with other psychotic and non-psychotic symptoms (11, 29–31). In light of this and, possibly, the growing influence of American psychiatry and mechanistic cognitive neuropsychiatry, the term pseudohallucination has been gradually falling into disuse so that it is not even referred to in the last version of the *Diagnostic and Statistical Manual of Mental Disorders* [DSM-5, (8)].

The concept of pseudohallucination is not historically univocal as it has been used with different meanings in the history of its existence. Here, Karl Jaspers' characterization of the term became one of the most prominent and widespread uses within the scientific community until very recently [see (28, 32, 33)]. Roughly speaking, the German psychiatrist proposes a distinction between genuine hallucinations and pseudohallucinations by predominantly appealing to the location of the voice and, secondarily, their sensory richness [See Jaspers (4, 34, 35)]. While real hallucinations occur in the “external objective space”, pseudohallucinations occur in the “inner subjective space”, establishing the inner/outer distinction as one of the main criteria for distinguishing between genuine hallucinations and pseudohallucinations (4). After exploring the use of the term pseudohallucination with a special focus on Jaspers' characterization, in this paper we examine how the inner/outer distinction remains an open challenge for neurocognitive theories about the origin of voices in schizophrenia. We propose that although the discriminatory value of the distinction has been systematically criticized at the empirical level, inner/outer considerations captured in first-personal phenomenological reports of auditory voices still need to be explained. We call this *the challenge from pseudohallucinations*. After exploring this issue in detail, we propose some phenomenological, conceptual, and empirical paths for future research. In this way, our paper is motivated by the idea that the inner/outer distinction posited by Jaspers can be rescued and integrated into a more dynamic and contextualized understanding of AVHs. We are using the inner/outer terminology to be purposefully broad and to capture a wide range of experiences and conceptualizations. We are aware that this distinction is ambiguous; in our paper however, we intend for the inner/outer distinction to mean voices inside/outside one's mental space and not just physical locality (e.g., confined within one's skull or in the space outside one's head or body). Although this distinction should not necessarily be used interchangeably with “genuine”- vs. “pseudo”-hallucinations, the latter concept still poses a significant challenge especially when it comes to the phenomenology of AVHs. There are also connotations about whether inner voices might be associated with a higher sense of agency, more controllability or even voluntariness, but evidence is inconsistent regarding the agential aspects of the inner/outer distinction.

## Distinguishing genuine from non-genuine hallucinatory voices

### A brief conceptual history of pseudohallucinations^1^

A quick analysis of the terms used by descriptive psychopathology reveals that a number of them are no longer in use, while others, although in current use, still produce several philosophical, empirical, and phenomenological problems. As noted by Berrios and Marková (36), the conceptual history of hallucinatory phenomena is long and complex. This historical process includes discussions about the representational vs. nonrepresentational nature of AVHs, the similarities and differences between hallucinatory and sensory perception, and the distinction (if any) between hallucinations found in psychiatric conditions vs. hallucinations found in purely neurological conditions, or even in general population. In this context, the use of the term pseudohallucination seems to emerge from a number of dichotomic formulations of multiple discussions regarding the nature of perception and imagination (4, 26, 35). Following the work of Esquirol (37), Falret (38), and Baillarger (39), the term pseudohallucination is introduced by Hagen (40) to designate general hallucinatory phenomena characterized by a lack of clarity and objectivity, with patients retaining awareness of their falseness. A clear antecedent of this notion is Michéa's (41) notion of *false hallucination* used to refer to those hallucinatory phenomena that are more than a mere idea or representation (non-sensory states) in the individual's stream of consciousness for they reveal vivid and defined objects; however, at the same time, such phenomena would be less than proper hallucinations as they will never impose themselves as real perceptions to the individual. On this view, the notion of pseudohallucination captures a type of phenomena located in the gray area between proper perceptual experience and hallucinations, or, in other words, phenomena that are neither real perceptions nor proper hallucinations. An initial examination of Michéa's view suggests that notions such as a sense of reality and sensory richness might be playing an important role in the distinction between AVHs and pseudohallucinations; However, there is no further analysis of this issue in his *Des Hallucinations*. Michéa's notion of pseudohallucinations will be systematically criticized by Berrios and Dening (32) due to internal theoretical inconsistencies.

Trying to develop an alternative and stronger framework, Kandinsky will distinguish real hallucinations from pseudohallucinations by referring to their objective quality. For Kandinsky (42), pseudohallucinations are “subjective perceptions similar to hallucinations, with respect to its character and vividness, but that differ from those because these do not have objective reality” (p. 134). Based on Kandinsky's work, Jaspers introduces his own definition of pseudohallucinations, a definition that will become one of the most influential within the psychiatric community for its practical value. Here, it is important to note that nineteenth Century's psychiatrists before Jaspers had already recognized the importance of phenomenological differences between voices heard *inside* and *outside* the head—along with the relevance of their variations in sensory richness—in distinguishing real hallucinations from pseudohallucinations. One example of this is Seglas' work (43) that refers to the term pseudohallucinations as the exacerbation of the internal verbalization of thought or *hyperendophasia*. The guiding idea within Seglas' view is that, internal verbalization of thought would become unknown to the person lacking external projection and preserving clarity and sensory richness, this explaining the phenomenology of pseudohallucinations as voices heard in the inner subjective space (p. 289–315).

### Jaspers' concept of pseudohallucinations

In a series of essays and in his *magnus opus*—*Allgemeine Psychopathologie*—Jaspers (4, 34, 35) establishes a sharp separation between AVHs and pseudohallucinations based on a more complex distinction between perception on the one side (*Wahrnehmung*), and “representation” or “image” (*Vorstellung*) on the other side. While perceptions generally enjoy the character of objective presence (*Leibhaftigkeit*), representations of objects have an imaginary-like quality (*Bildhaftigkeit*). In this sense, while real AVHs are experienced as objectively present (*leibhaftig*), pseudohallucinations are experienced as imaginary or subjective [see (29)]. Jaspers' work proposes that real hallucinations are false perceptions of an external objective reality without corresponding stimuli, while pseudohallucinations are special forms of imagination or representations [(35), p. 89], establishing an important separation between perception and purely representational (non-sensory) states^2^. It follows that real hallucinations have a corporeal, objective character, projected in the external space, while pseudohallucinations are imaginary, subjective and appear in the internal (or subjective) field of awareness. Recently, Knappik et al. (45) have proposed that research on inner vs. outer voices since the 1970s has been profoundly affected by a conceptual confusion between very different readings of this distinction. On the one hand, a reading in terms of *apparent location of voices*, is literally understood as inside or outside the skull of the individual. On the other hand, a metaphorical reading where ‘outside my head' means apparently real, while “inside my head” actually means that it is phenomenally apparent to individuals that the voices aren't real. For the authors of the current paper, authors such as Kandisky and Jaspers use the metaphorical reading. We believe that this distinction might be orthogonal to the way we examine Jaspers here. For Jaspers, perceptions are complete, enjoy sensory richness, are easily retained, and are independent of the individual's will. However, it is essential to point out that, according to Jaspers, what separates perception from representation or “images” through an abyss is the *corporeality* and *exteriority* of the former so that the representations can successively acquire all the other characteristics that have been attributed to the perceptions. In other words, it is important to consider that the property of location is used in interaction with other properties to define the limitations of the distinction between proper AVHs and pseudohallucinations^3^. Nevertheless, Jaspers' notion of outer space or exteriority may well be a metaphorical one: he acknowledges pseudohallucinations that are physically located in outer space, and genuine hallucinations that are physically located inside the body [(45), p. 222]. Furthermore, apparent physical location, apparent reality and sensory richness are not synonyms. These features may be varying in degree, quality and intensity, sometimes in a mixed-up or even paradoxical fashion. For example, a patient with chronic schizophrenia taking antipsychotic medications might still hear highly perceptualized and external/outer voices in terms of perceived location (outside one's mental space) but does not consider them to be real and may well explain them as “hallucinations” or “just voices”.

Phenomenological analysis and direct clinical observation show that patients' reports on voices do not easily fit into the categories proposed by Jaspers when trying to distinguish genuine AVHs from pseudohallucinations [see for example (46–48)]. It is not clear if distinctions between “genuine” and “pseudo” phenomena really stand in light of the phenomenological heterogeneity of auditory verbal hallucinatory phenomena. In the same way that a member of a certain family, failing to instantiate certain features commonly associated to his or her relatives (spontaneity, loud talk, cheeriness, and the like) can still belong to the family, phenomenological heterogeneity regarding inner/outer distinctions and sensory richness might not be sufficient for positing a separate phenomenon. This issue becomes even more difficult if one considers psychotic symptoms as evolving and flexible states over time where inner/outer distinction might not really capture genuine/pseudo differences, but rather, different stages of their evolution or different combinations and interactions between different aspects of the individual, the environment, and the phenomenon. About this, it has been observed that surveys on the phenomenology of voices show that they are perceived with varying degrees of sensory richness, objectivity, internal or external projection and insight in a continuum over time (1, 10, 14). Such heterogeneity makes it really difficult to posit sharp distinctions between genuine AVHs and pseudohallucinations as clearly *distinct* phenomena based on inner/outer and sensory richness. Furthermore, under certain characterizations, this distinction might make pseudohallucinations a type of purely cognitive experience (non-sensory) which, in turn, is not consistent with the phenomenological reports. More problematic is that Jaspers' hallucination-pseudohallucination distinction may lead to an unwarranted claim, namely, that voices with genuine hallucinatory characteristics are exceptional in schizophrenia. As mentioned in the introductory section, a number of studies reflect a wide heterogeneity in the descriptions of the patients' voices. While some people report phenomenal clarity similar to a conversation with another person (49), others report them as ideas or “silent sensations” (16, 21). Most patients manage to differentiate voices from their own thoughts (30, 49) and they usually have normal volume. Although many patients hear more than one voice, most refer to a middle-aged male voice that commands or insults but may also make positive comments (20). Less frequent are whispers or shouts, and, very importantly for our discussion, voices are referred as both inside and outside the head (16, 21, 23).

Despite all the aforementioned variations in the richness of phenomenological descriptions and the temporally dynamic nature of first-personal reports, Stephane et al. (9) concludes that auditory hallucinatory phenomena in people with schizophrenia can be organized into two clusters. Applying Hierarchical Cluster (HC) and multidimensional scaling (MDS) analyses to twenty phenomenological variables (each one of them with a binary value assigned), Stephane et al. (9) describes (i) a first cluster of verbal hallucinatory phenomena with low linguistic complexity, repetitive content, self-attributed, located in the external objective space and associated with control strategies and, a (ii) second cluster of hallucinatory phenomena with high linguistic complexity, systematized content, multiple voices, attributed to others and located in the internal subjective space. Although Stephane's view seems to incorporate Jaspers' inner/outer distinction into a broader categorization of hallucinatory voices, this taxonomy might fail to include cases of voices with high linguistic complexity located in the external world.

In any case, the incorporation of the inner/outer distinction into the phenomenological heterogeneity and temporally evolving nature of auditory verbal hallucinatory phenomena is a strategy we have openly supported (28). According to Docherty et al. (50), patients with internal hallucinations (experienced in the internal field of awareness like the ones described by Jaspers as pseudohallucinations) do not differ from those with external hallucinations on the severity of other symptoms. However, they reported their hallucinations to be more emotionally distressing, longer-lasting, less controllable, and less likely to remit over time. This study also reports that patients were also more likely to experience voices commenting, conversing, or commanding, and to have insight into their voices' self-generated nature. An important thing to note here is that individuals with internal hallucinations were not older but had a later illness onset (50). Docherty's et al. findings might open the pathway to exploring the semiologic and clinical value of the inner/outer distinction in the understanding of AVHs.

Importantly, a generally ignored fundamental observation in this context is that, in the same individual, the many properties of voices can change according to their clinical state and evolution over time [this is already noted by (51)]. In fact, the same individual's reports over time might change due to a number of reasons, among them, the impact and effectiveness of pharmacological treatment, psychotherapeutic treatment, anxiety levels, personal resources, and attributional styles (52, 53). About this, Sterzer et al. (54) claims that the lack of recognition of the temporally evolving nature of psychotic symptoms might be a weakness of current—more static—approaches within the field. In this sense, we believe that the integration of the inner/outer distinction might help to broaden our current views about auditory verbal hallucinatory phenomena. Certainly, more research considering the temporal evolution of psychotic symptoms in the same individual would enlighten our target debate.

## Rescuing Jaspers' inner/outer distinction: The challenge from pseudohallucinations

The inner/outer distinction posited by Jaspers' characterization of the concept of pseudohallucination should not be ignored. It is a source of valuable insight for it constitutes relevant phenomenological *data*. As reported, qualitative features of hallucinatory phenomena in schizophrenia are gradual and temporally dynamic, and sharp and static distinctions fail to capture this fact. As Stephane (23) suggests, considerations about the phenomenology of hallucinatory voices in schizophrenia are not merely a pointless curiosity, but rather, they might inform the construction of specific categories to be studied from an experimental point of view [see also (55, 56)]. It is very important to stress the fact that inner/outer distinctions appears in first-personal reports of auditory hallucinatory phenomena, so they might reflect a dimension of such conscious experience worth exploring from phenomenological and empirical points of view (2, 4, 16, 29). In this respect, *radical eliminativist alternatives* about the term pseudohallucinations should be taken with caution. Such options might lead us to the neglect of relevant phenomenological information about the subjective structure and dynamic nature of AVHs, and, as a consequence, they might prevent us from integrating important elements into neurobiological explanations for the origin of hallucinatory voices. For this reason, moderate options should be preferred. As it has been suggested, precise and accurate analysis of first-personal phenomenological reports is fundamental in order to establish a clear *explanandum* for potential neurobiological theories of psychopathological phenomena (57–60). Here, even if for some, the genuine/pseudo distinction posited by Jaspers might not be useful to distinguish *distinct* phenomena at the neurobiological level, inner/outer considerations might help to broaden and add phenomenological complexity to theories aiming at explaining the dynamic and interactive nature of hallucinatory phenomena.

Here we see a clear opportunity for phenomenological distinctions to provide more precise ways to look into the brain and the complexities of conscious experience in the study of altered states of consciousness such as hallucinatory voices. It might be true that, in light of the current available empirical evidence, pseudohallucinations do not constitute a totally distinct phenomenon at the neurobiological level, however, inner/outer phenomenological differences in reports need to be explained at this level as well. The exploration of this issue might help us to understand how the ways in which verbal hallucinatory phenomena interact with other environmental, neural, structural, and psychological features of the individual over time might be reflected in the first-personal phenomenological reports. We believe that strong distinctions between genuine hallucinations and pseudohallucination *as distinct phenomena* based on restrictive criteria might not only present a decontextualized picture of the phenomena, but also, they might not be able to reflect their dynamic and temporally evolving nature. Arguably, the potential transition from inner to outer voices (and *vice versa*) might be an example of such a temporally dynamic and multifactorial nature of general auditory verbal hallucinatory phenomena, and perhaps, a *moderate view* might defend the elimination of the genuine/pseudo distinction, while preserving the inner/outer consideration. Here, however, we are left with an important challenge: even if we incorporate hallucinatory phenomena experienced in the inner space into the diversity of general auditory verbal hallucinatory phenomena, we still need to explore what underlies such a phenomenological distinction at the neurocognitive level. We explore this issue in the following section.

## Dealing with the challenge from pseudohallucinations

### Inner speech, auditory verbal hallucinations, and the inner/outer distinction

Published research in the field points toward three main potential neurobiological origins for AVHs: alterations in thought, memory, or spontaneous epileptic-like activations of the auditory cortex. In this context, the hypothesis of perceived thoughts or inner speech has become a natural place to look as AVHs are often referred to as *perception of speech* [cf. 23, (56, 61, 62)]. Inner speech has been similarly characterized as the silent expression of thoughts in a coherent linguistic form (63, 64), as the silent production of words in one's mind, or as the activity of talking to oneself in silence (65). Importantly, inner speech has been often regarded as the fundamental element to a number of states and processes underlying our mental life such as consciousness, self-awareness and self-regulation (66).

The inner speech hypothesis of AVHs should be consistent with the phenomenological reports. In order to understand how inner speech may acquire the type of sensorial and locational properties characteristic of such reports, some authors have investigated the description of the relationship between thoughts and language pioneered by Vygotsky (63). The author claims that mental functions appear twice in development. First, as an *interpsychological activity* (that is, taking place between more than one individual), and secondly, *intrapsychologically*, resulting from an internalization process (67). Here, thought would be the transformation of external dialogue into internal dialogue. According to Vygotsky, original external speech undergoes important syntactic and semantic changes losing most of its acoustic and structural qualities and becoming finally “thinking in pure meanings”. The basis for the hypotheses concerning the perception of inner speech as the origin of AVHs points toward a failure in inhibitory mechanisms in the brain (68). However, even if true, this hypothesis might not explain the origin of thoughts' sensorial qualities needed to relate inner speech and auditory verbal hallucinatory phenomena as a plausible explanatory framework. Non-monitored thinking activity might only explain certain thoughts popping into an individual's stream of consciousness as unexpected or decontextualized (69). Three positions trying to address this issue can be identified in current literature. First, Oppenheim and Dell (70, 71) propose that inner speech is similar to overt speaking but only lacking the production of sound. Second, Abramson and Goldinger (72) suggest that inner speech is processed without articulatory information and, third, Stephane et al. (56) proposes that inner speech and thought are processed by entirely different neural mechanisms. This latter idea might actually overcome some conceptual problems addressed by López-Silva (69) and Proust (73) when exploring the parallelism established between motor action and thought in current neurobiological research in psychiatry, especially delusions.

Although these issues are the focus of intense discussion in the current literature, a number of authors tend to claim that most current empirical evidence points toward the idea that inner speech is associated with the activation of both language production and perception areas in the brain, and a number of authors also agree that inner speech has semantic and auditory-phonological components (74). Based on Vygotsky's work, Fernyhough (75) suggests that alterations of the transformation that social speech undergoes to become inner speech could result in inner speech attributed to an other. However, as Stephane (23) rightly points out, the exploration of inner-speech-based hypotheses focus on explaining self-generated inner speech to others, and remain unclear about the location of the resulting conscious experience.

Recently, Langland-Hassan (74) raises the possibility that inner speech is a construct that comprises more than one process serving multiple functions. Some of these processes—such as working memory and the internal rehearsal of overt speech—might require the *representation* of the acoustic properties of language, while others, like automatic pre-reflective thinking, would have a purely semantic and amodal nature. In this sense, Vygotsky conceives different levels of thinking with a progression in complexity and conscious control. A potential alternative is that what we call AVHs are the endowment of acoustic qualities to normally impoverished forms of inner speech *via* faulty processing. These thoughts—normally out of conscious control—would then seem alien to the individual (76). However, further research is needed in order to provide support to this hypothesis. In contrast, Gauker (77) argues that inner speech itself ought not to be confused with auditory verbal imagery, just as one distinguishes between one's own auditory perception of another's speech and that speech itself. Gauker proposes that one should distinguish between the representation of one's own inner speech—which takes place through the use of auditory verbal imagery—and inner speech itself, which lacks any sensory component. On Gauker's view, inner speech would be non-sensory in nature, so it is still an open challenge to explain how inner speech might produce the sensory qualities of AVHs.

As we can note, current models based on inner speech alterations as the origin of AVHs still need to clarify the way in which inner speech itself might acquire sensorial properties such as volume and location commonly present in the phenomenological reports of voices in schizophrenia. Without a view on this issue, inner speech-based approaches still face an important explanatory challenge when trying to make sense of the origin of AVHs in general, and the inner/outer distinction specifically. Very recently, Stephane et al. (56) used fMRI to study the activation of the “where” auditory pathway during silent and aloud reading in order to investigate the neural basis for inner speech. Localization of inner and external voices in internal and external space, respectively, depends on the activity of the “where” auditory pathway. Therefore, dysfunction of this pathway could result in the experience of the inner voice in external space, which could account for the outer perception of inner speech as occurs in AVHs and possibly other psychotic experiences such as thought broadcasting and mind reading. Recent studies show that inner speech abnormalities in AVHs also involve spatial externalization (hearing one's own inner voice outside the head) and that agency and spatial externalizations are independent at phenomenological and cognitive levels and are co-related across levels (24). For Stephane et al. (56), these externalizations could reflect a dysfunction of independent neural networks, thus pointing toward the “where” auditory pathway as a candidate in the case of spatial externalizations. However, it is not clear how this model explains why hallucinatory voices heard in the inner space are experienced as not produced by the individuals themselves, which is something observed in the phenomenological studies of AVHs reports. Stephane et al. (56) suggest that while inner speech has been often examined in hallucinations [see (78)], the internal space localization of the inner voice in patients with AVHs has not been investigated yet. We suggest that this line of research might help to clarify how to integrate the inner/outer distinction present in the phenomenological surveys about the experience of AVHs into the theories appealing to inner speech alterations when exploring the neural basis of the phenomenon.

### The neurobiology of hallucinatory voices in schizophrenia and the inner/outer challenge

Multiple neurobiological models attempt to explain AVHs in the specific case of schizophrenia. One of the most popular approaches posits a failure in inhibitory processes in the auditory cortex, leaving the sensory stimuli associated with thoughts unsuppressed, and therefore, perceptible to the individual. This hypothesis can be traced back to Feinberg (79). On this view, thought has been considered as our most complex motor act (80), generated by the same computational and integrative mechanisms of speech [(79); for a criticism of this idea see (69, 73)]. Normally, the frontal lobe sends an inhibitory signal to the auditory cortex (corollary discharge), minimizing the perception of the sensory reafference of the individual's own speech (81). This mechanism has been found to be deficient in schizophrenia, independent from the individual's expectation or feeling of agency (82).

In support of the existence of an abnormal generation of corollary discharges, comparisons of patients with schizophrenia and control groups in different conditions (silent thinking, talking, listening to own speech) show a dampening of auditory cortex responsivity during talking and inner speech in control groups but not in patients with schizophrenia (83). From a structural perspective, a widespread reduction in white matter fractional anisotropy has been reported both in chronic and recent-onset schizophrenia (84). Decreases in fractional anisotropy have been reported in tracts connecting medial and ventral frontal regions with posterior parietal or medial temporal areas (85). However, there are also findings of preserved and increased anisotropy in the left *arcuate fasciculi* (86). In this context, an unresolved question for the proposition of a faulty corollary discharge as the origin of AVHs has to do with the origin of the auditory reafferences in the case of unuttered thoughts.

An expanded version of the abovementioned approach considers inner speech as part of a more extensive monitoring system for motor activity, including efference copies, forward models, and comparators (87). Inner speech could be considered as the sensory output of a forward model that serves as a prediction of the anticipated sensory input of planned overt utterances, and what we experience as inner speech—in the absence of overt speech—might simply be this speech prediction *running offline*. Existing neuroimaging evidence provides validation of some of the conceptual tenets of this model, such as the idea that both inner and overt speech production involves generating a sensory prediction (88, 89). The auditory cortex is not the only region that has been related to AVHs in schizophrenia. Waters and Jardri (90) have argued that the associative, rather than the primary auditory cortices, are regularly activated during AVHs. Jardri et al. (91) examined several functional neuroimaging studies of AVHs and conclude that the experience of AVHs is associated with increased activity in frontotemporal areas involved in speech generation and speech perception, but also within the medial temporal lobe, a structure notably involved in verbal memory. In this sense, language areas in the temporal lobe overlap with the temporoparietal junction, and this region has been attributed a critical function integrating motor and sensory aspects, or, in other words, the representation of speech.

Regarding differences between internally and externally perceived hallucinatory voices, convergent anatomical differences were detected between the patient subgroups in the right temporoparietal junction (rTPJ). Compared to healthy individuals, opposite deviations in white matter volumes and sulcus displacements were found in patients with inner space hallucination and patients with outer space hallucination (92). Current results indicate that AVHs' spatial location is associated with the rTPJ anatomy, a key region of the “where” auditory pathway. The temporoparietal junction plays a crucial role in the localization of language and it is part of a system for processing social cognition, emotion, self-representation, and agency. The inferior parietal and posterior superior temporal regions contain multi-modal representational systems that may provide rapid feedback and feed-forward activation to unimodal regions such as the auditory cortex. It has been proposed that the over-activation of these regions could not only result in erroneous activation of semantic and speech (auditory word) representations, but also in processes related to alterations in theory of mind, and problems for establishing boundaries between self and non-self. However, as the internal space localization of inner speech in patients with hallucinations has not been specifically investigated, the challenge seems to remain open in this context.

## Situating verbal auditory hallucinatory phenomena within context: Potential paths for future research

We have suggested that even if the genuine/pseudo distinction applied to hallucinatory voices is not able to capture distinct phenomena at the neurobiological level, inner/outer distinctions present in the phenomenological reports of AVHs remain an open explanatory challenge. We have called this “the challenge from pseudohallucinations”. We have proposed that inner/outer phenomenological differences might be a sign of the dynamic and flexible nature of general auditory verbal phenomena. As aforementioned, hallucinatory voices heard in the inner space have been described as more distressing, less controllable, and less likely to remit than when compared with those heard from the outside (50). These clinical features make the integration of the inner/outer distinction into the explanatory framework of AVHs not a mere conceptual curiosity, but a need with potential clinical implications. However, such a task is not simple. In this section, we sketch some phenomenological, conceptual, and empirical issues we should take into consideration in order to make sense of the inner/outer consideration when understanding auditory verbal hallucinatory phenomena.

### Auditory verbal hallucinations in its phenomenological context

First of all, hallucinatory voices in schizophrenia emerge in a phenomenologically altered context (31, 52, 93–96). Arguably, inner/outer differences might be linked to some specific interactions between neurobiological, psychological, and phenomenological elements of the subjective experience of hallucinatory voices and more general alterations in perception and embodiment. Commonly, approaches to AVHs within the neurocognitive tradition have focused on the description of the subjective structure of the immediate experience of *hearing voices* (10, 13). Although we have already recognized the fundamental relevance of this type of approach, there is a way in which phenomenological analysis might be expanded, and, with it, a more contextualized picture of the phenomenon might be constructed in order to inform more specific experimental work (a desirable feature of scientific *explananda*). As suggested by Raballo (31), voices in schizophrenia involve a global transformation of the relationship between the self and the world, where the “felt-naturalness of the psychic field seems changed” (p. 137). As pointed out by Mishara and Zaytseva (29), overly mechanistic views within neurocognitive sciences might miss the contextual phenomenological complexity in which AVHs arise, and with this, a number of key perceptual transformations that accompany immediate experiences of hallucinatory voices might be neglected [see also (48, 55)]. We believe that the construction of a contextualized and dynamic *explanandum* for neurocognitive theories of AVHs might benefit from this broader type of phenomenological examination. However, on this view, AVHs are not only a sign of changes in the quality of specific sensory modalities, but also, they point toward a more general change in the structure of the entire field of consciousness. Hallucinatory voices emerge in a phenomenologically complex context that goes beyond changes in specific sensory modalities (97) and therefore, the popular definition of AVHs as *perceptions of speech in the auditory modality without corresponding external stimuli* might be too restrictive. Here a more granular and contextualized phenomenological approach to hallucinatory voices is required and we believe that the observations provided by the Early Heidelberg School of Psychiatry might help to advance this task.

As observed by Mayer-Gross and Stein (76, 98), AVHs are symptomatic of more general low-level perceptual abnormalities very difficult to articulate for patients because they are unlike any prior everyday sensory experience. Mayer-Gross openly opposes Jaspers' strong distinction between genuine and pseudohallucinations and assumes a more dynamic view of hallucinatory phenomena [see (29)]. For Mayer-Gross, low-level perceptual abnormalities underlie fundamental transformations of perception in its various modalities that may involve a general disruption in the timing of the perception-action coupling (99, 100). Empirical research on this issue suggests that anomalies of temporal processing in schizophrenia patients emerge at very brief time scales [8 and 17 ms; (101)]. About this, Pienkos et al. (48) suggest that protentions—often non-conscious anticipations that serve as the context for moment-to-moment predictions—are altered in psychosis and occur very early in the “phenomenological hierarchy” just as proposed by Mayer-Gross. All these low-level perceptual alterations in the factory of conscious reality would lead to a complete variation of the *Gestalt*-Structure of experience, the pervasiveness of these alterations also leads to a general loss of perspective due to the disruption of the embodied relationship between the self and the objects of perception (102, 103). This is consistent with what Blankenburg observes in patients with schizophrenia, namely, that symptoms such as AVHs are often experienced in connection with abnormal bodily sensations, especially in the case of those experienced as “made” from outside.

These generalized changes in perception and embodiment seem to underlie a loss of perspective where patients with schizophrenia show difficulties in integrating multimodal information effectively (104). Importantly, Mishara and Zaytseva (29) have recently suggested that this loss of perspective may be more characteristic of hallucinations in schizophrenia. Here it is important to note that, for Mayer-Gross, hallucinations do not appear in a phenomenological space comparable to normal perceptions. Rather, hallucinatory phenomena occur in a highly salient fragmentary space when compared to other contingent objects in the field of awareness, hijacking the individual's attention. Mayer-Gross indicates that while non-psychotic individuals can correct their perspective once they try to engage with the hallucinatory object, individuals with schizophrenia show difficulties in adapting their perspectives when trying to explore their hallucinatory states.

In order to explore how this view might inform the integration of inner/outer considerations in the study of AVHs, we need to consider that the functional change in the Gestalt organization of the experience usually happens at different levels (105–107). Conscious experiences are characterized by transitions between different levels of consciousness, where self-organizing embodied awareness might change. Here, inner/outer reports should be examined by taking into consideration these more general perceptual transformations underlying the hallucinatory reality of individuals. Arguably, inner/outer considerations might help us to understand how hallucinatory voices interact with a generally diminished embodied and cognitive performance in patients with schizophrenia. Importantly, it has been suggested that AVHs tend to overlap with delusions of reference, persecution, control, thought insertion, and thought withdrawal (14, 48). All these symptoms show a permeation of ego boundaries that might be related to inner/outer shifts in perspective. Hypothetically, inner/outer distinctions might also mean that one's consciousness is making efforts to maintain Gestalt coherence within a generally altered perception-action cycle. Due to the inability to integrate other types of perceptual and cognitive multimodal information, and the generalized loss of perspective, inner/outer considerations might suggest a generalized alteration in the relationship between the hallucinator and the hallucination. Integrative research on this issue might help to open broader approaches to the understanding of AVHs and their relationship with other symptoms such as delusions.

Mayer-Gross' observations have been recently supported by a number of empirical studies that found evidence of fragmentation and changes in perceptual organization in schizophrenia. These changes include difficulties seeing objects or scenes as whole gestalts (108, 109). In the same line, Fuchs (110) observes in patients with schizophrenia alterations in the ability to link the present moment with what is about to happen, and to move into the probable or anticipated future, which would be consistent with the idea of a general disconnection and fragmentation of perceived events underlying hallucinatory phenomena. About this, Silverstein et al. (111) conclude that perceptual fragmentation may contribute to figure-ground confusion and loss of perceptual stability, with objects appearing to change shape or appearance. This type of evidence provides support for the idea that sensory and perceptual low-level anomalies such the ones described by the Early Heidelberg School, including perceptual disorganization, may be implicated in hallucinations. Certainly, further investigations of these changes and the processes involved may shed light on the specific development of AVHs and their specific phenomenological features.

### Auditory verbal hallucinations as the product of top-down and bottom-up alterations

The aforementioned phenomenological considerations also provide a fundamental conceptual insight. One of the main problems underlying most neurocognitive models of AVHs is the implicit reproduction of the idea that representational states (such as beliefs, thoughts, imaginations, etc.) are totally distinct and widely separated from perceptual states. This assumption clearly resonates with the distinction posited by Jaspers between perception on the one side (*Wahrnehmung*), and “representation” or “image” (*Vorstellung*) on the other side that serves as the theoretical foundation for his distinction between genuine AVHs and pseudohallucinations. A lesson to be learnt here is that, perhaps, the very conceptual picture of the relationship between representations or “images” and perceptions as distinct and separate phenomena underlying our target debate provides us with an oversimplified approach to the formation of hallucinatory phenomena. Arguably, representation and perception are not so distant from each other in the process of creating conscious experience. Certainly, the study of AVHs might benefit from the adoption of a less static and modular view of perception and consciousness, which in turn might help to clarify the relationships (overlapping presence and initial distinguishability) between the origins of hallucinatory voices and delusions of a different kind. Approaches positing alterations in both representational and perceptual states altogether might be able to offer a better understanding of the origin of AVHs and their evolution over time by including bottom-up (perceptual) and top-down (cognitive) alterations. Research on the neurobiological origin of voices in schizophrenia has predominately pointed toward alterations in the auditory cortex, formulating the phenomenon as having a predominant bottom-up perceptual nature (23). However, here we find two open problems. First, current neurobiological approaches might not be able to explain the presence of non-self-attributed voices experienced in the inner space. Second, alterations in the temporoparietal junction have also been found in hallucinatory states, suggesting problems in the production and organization of representational states, an issue that should be included into theoretical explanatory models of AVHs as having a potential top-down component. In this sense, strong priors have been regarded as playing a fundamental role in hallucination pathogenesis (112). From a strictly neurobiological point of view, however, clarifying whether the inner/outer distinction truly reflects two different mechanisms at the neural level could have implications for the current understanding of the origin, evolution and treatment of the condition.

Furthermore, the addition of a top-down component into the understanding of AVHs might help to clarify the temporally evolving nature of hallucinatory phenomena. Top-down expectations and beliefs might interact in different ways with the type of altered perceptual inputs described underlying hallucinatory realities (112). Inner/outer differences might also be the result of that interaction. Very recently, contradicting several earlier studies using the Symptom Capture Paradigm (78, 113), Fuentes-Claramonte et al. (114) found no evidence in their study suggesting that the experience of AVHs in schizophrenia is associated with auditory cortex activation. For the authors, coupled with the fact that perception of formally similar real speech strongly activated a large expanse of the superior temporal cortex, this failure would seem to exclude theoretical approaches to AVHs in schizophrenia that invoke abnormal neural activity in the auditory cortex, opening the possibilities for cognitive-based approaches to the phenomenon. It is not the purpose of this paper to extensively review and evaluate the plausibility of such strong conclusions (which we think might have insufficient empirical support); however, we should point out that the evidence provided by Fuentes-Claramontes et al. (114) still might not be able to make sense of the sensorial nature and the inner/outer distinction present in first-personal reports of AVHs. If AVHs are cognitive in nature, they still have to explain how those states acquire all the sensory features expressed in first-personal reports, including the existence of inner and outer voices, and, from this point of view, the observations of the Early Heidelberg School are still relevant. Considering that over the last years a number of authors within cognitive sciences have been proposing that the distance between cognitive (beliefs, thoughts, imaginations, etc.) and perceptual states in the process of producing our conscious experience of reality might not be so wide as thought by Jaspers. In other words, cognitive and perceptual states are closely intertwined in the process of producing our experience of reality. Specifically, Sterzer et al. (54), Kargel et al. (115), among many others, have suggested that hallucinatory phenomena might be studied using the *predictive coding framework*. On this view, psychotic symptoms arise from a dysfunction in the interaction between top-down expectations and bottom-up information. The idea is that the feedforward-modeling characteristic of the brain continuously expecting perceptual inputs based on prior states that are already present and the sensory input from external environment produces the experience of reality. A predictive coding account provided by Sterzer et al. (54, 116) tries to reconciliate top-down and bottom-up alterations in individuals with AVHs and delusions in schizophrenia. Although this model might be a potential starting point for connecting representations and perceptions into a more complex and dynamic view of the formation of conscious states, it is still an open challenge for the way in which it might be able to make sense of the inner/outer distinction observed in the phenomenological reports of AVHs.

## Concluding remarks

The concept of pseudohallucination is originally introduced in the context of European psychiatry to designate hallucinatory-like phenomena not exhibiting some of the paradigmatic features of “genuine” hallucinations. After its introduction, Karl Jaspers locates the notion of pseudohallucinations into the auditory domain, appealing to a distinction between hallucinatory voices heard within the subjective *inner* space (pseudohallucination) and voices heard in the *outer* external space (real hallucinations) with differences in their sensory richness. Over the last years, the notion of pseudohallucination has been the target of a number of criticisms, one of the most important being its inability to distinguish distinct phenomena at the neurobiological level. We have argued that that even if the concept of pseudohallucination is not helpful to differentiate *distinct* phenomena at this level, the inner/outer distinction highlighted by Jaspers' characterization of the term still remains an open explanatory challenge for dominant theories about the neurocognitive origin of AVHs. We have called this “*the challenge from pseudohallucinations”*. Here, it is important to note that apart from distinguishing the voices in an inner or outer space, patients describe multiple characteristics associated with their hallucinations. So far, none of these seems to match the theoretical models or the scientific findings unequivocally. However, there might be multiple associations of neural events and personal elements resulting in individual hallucinatory experiences that are phenomenologically diverse across individuals. Top-down elements such as prior beliefs, attributional and semantic factors might also modify the communication of the individual's subjective experience. From this point of view, it might be suggested that hallucinatory voices mutate during the schizophrenic condition, potentially reflecting different stages in this evolution. One of the best descriptions of hallucinatory voices is found in Kraepelin's work. According to this author, patients begin by hearing noises or sounds, often confused with external stimuli. Later, the voices appear inside their heads. This is similar to prodromal symptoms (whistling, applause, slamming doors, etc.) typical of people at high risk of psychosis. Even if a single neurobiological or cognitive mechanism cannot explain all the voices in schizophrenia, attention needs to be paid to the individuals' descriptions, and the way in which the reports change over time in a single individual. We hypothesize that the possibility that certain phenomenological characteristics of AVHs could have clinical and semiologic values orienting toward specific brain, cognitive, and phenomenological abnormalities, although perhaps not directly and categorically, but instead, in a more dimensional way. While for some authors hearing voices outside the head would be a more serious issue in terms of the severity of the underlying dysfunction, others do not confirm this observation (50). We have also proposed that an analysis of the altered perceptual context in which hallucinatory voices emerge might open potential paths to making sense of inner/outer differences from a phenomenological point of view. Such observations would also necessitate the adoption of more dynamic and temporally evolving approaches to perception and psychotic symptoms that are able to integrate the inner/outer distinction in a more complex and contextualized view of auditory verbal hallucinatory phenomena. Finally, we have suggested that the inclusion of top-down and bottom-up elements in the understanding of perceptual experience might also help to guide future empirical explorations of the origin of AVHs and their overlap with other negative and positive symptoms such as delusions.

## Data availability statement

The original contributions presented in the study are included in the article/supplementary material, further inquiries can be directed to the corresponding author.

## Author contributions

PL-S and ÁC were involved in data analysis and writing. CH was involved in data analysis and corrections. All authors contributed to the article and approved the submitted version.

## Conflict of interest

The authors declare that the research was conducted in the absence of any commercial or financial relationships that could be construed as a potential conflict of interest.

## Publisher's note

All claims expressed in this article are solely those of the authors and do not necessarily represent those of their affiliated organizations, or those of the publisher, the editors and the reviewers. Any product that may be evaluated in this article, or claim that may be made by its manufacturer, is not guaranteed or endorsed by the publisher.

## Footnotes

^1^In order to select the relevant literature for our analysis, a computerized search in both Spanish and English (period 1990–2021) was performed in the following databases: Clarivate, PsyINFO, Medline, Scopus, Scielo, Redalyc, Elsevier, Erih+, The Philosopher's Index, and Jstor. The terms included were: (English) “auditory verbal hallucinations,” “pseudohallucinations,” “schizophrenia,” (Spanish), “halucinaciones auditivas,” “pseudoalucinaciones,” and “esquizofrenia.” The quality and relevance of the main source of information - peer-reviewed articles - were assessed through the editorial criteria and impact-factor provided by the databases from which they were retrieved. This source was complemented by (i) the review of classic books on the topic, (ii) peer-reviewed articles cited by the primary sources and, (iii) the review of diagnostic manuals. No major technical differences were found between descriptions of the phenomenon in Spanish and English publications.

^2^For a discussion on this distinction, see: Bayne and Montague (44).

^3^We thank the reviewers for noting this issue. A full assessment of the impact of this re-examination on empirical research would take us too far from the main aim of this paper. However, we believe that this is a necessary task for future work in the field.
